# Long non-coding RNA AFAP1-AS1 facilitates tumor growth and promotes metastasis in colorectal cancer

**DOI:** 10.1186/s40659-016-0094-3

**Published:** 2016-08-30

**Authors:** Xu Han, Lingling Wang, Yu Ning, Shuang Li, Zhenjun Wang

**Affiliations:** 1 General surgery, Chao-Yang Hospital of Capital Medical University, 8 Gongtinan Rd, Beijing, 10001 People’s Republic of China; 2 Anorectal surgery, Beihua University, Jilin, 132001 People’s Republic of China; 3 Department of Neurology, Beihua University, Jilin, 132001 People’s Republic of China

**Keywords:** AFAP1-AS1, Colorectal cancer, Tumor metastasis, Epithelial–mesenchymal transition (EMT)

## Abstract

**Background and objective:**

Long non-coding RNAs can regulate tumorigenesis of various cancers. Dys-regulation of lncRNA-AFAP1-AS1 has not been studied in colorectal carcinoma (CRC). This study was to examine the function involvement of AFAP1-AS1 in tumor growth and metastasis of CRC.

**Methods:**

Relative expression of AFAP1-AS1 in CRC tissues and CRC cells lines was determined using quantitative real-time PCR (qRT-PCR). Functional involvement of AFAP1-AS1 in tumor proliferation and metastasis was evaluated in AFAP1-AS1-specific siRNA-treated CRC cells and in CRC cell xenograft. Expression of epithelial-mesenchymal transition (EMT)-related gene expression was determined using western blot.

**Results:**

Relative expression of AFAP1-AS1 was significantly elevated in CRC tissues and CRC HCT116 and SW480 cell lines. AFAP1-AS1 knock-down suppressed SW480 cell proliferation, colony formation, migration and invasion. Also AFAP1-AS1 knock-down inhibited tumor metastasis-associated genes expression in terms of EMT. This carcinostatic action by AFAP1-AS1 knock-down was further confirmed by suppression of tumor formation and hepatic metastasis of CRC cells in nude mice.

**Conclusion:**

lncRNA-AFAP1-AS1 knock-down exhibits antitumor effect on colorectal carcinoma in respects of suppression of cell proliferation and metastasis of cancer cells.

## Background

Colorectal carcinoma (CRC) is the third cause of cancer incidence and death worldwide [1]. Number of CRC patients is increasing in China. Although treatment methods have been improved, CRC still cannot be cure. Death occurs because of metastasis to other organ [2]. Elaboration of molecular pathogenesis underlying CRC may provide new therapeutic strategy.

Long non-coding RNAs (lncRNAs) are kind of RNA molecule with a length of longer than 200 nucleotides and have no ability of coding protein [3]. As existing research results suggested that several lncRNAs are involved in transcriptional regulation, organization of nuclear structure, and post-transcriptional processing. Increasing evidences suggest that lncRNAs are involved in a larger range of cellular biological processes, including growth, proliferation and differentiation. Dysregulation of lncRNAs has been preliminary proved to contribute to carcinogenesis, invasion, and metastasis of various cancers [4–6]. However the function of most lncRNAs remains unclear. The identification of cancer-related lncRNAs and investigation of their molecular pathogenesis basis in cancers are important.

One lncRNA, actin filament associated protein 1 antisense RNA1 (AFAP1-AS1), was the most significantly upregulated in pancreatic ductal adenocarcinoma, esophageal adenocarcinoma and lung cancer, and associated with poor prognosis [7–9]. However the correlation and functional involvement of AFAP1-AS1 in CRC remains unclear. The potential function of AFAP1-AS1 in cancers is its regulatory role in tumor growth, invasion and metastasis. Tumor metastasis is a process of multiple gene regulation cascades. Epithelial-to-mesenchymal transition (EMT) serves as a common biological mechanism underlying tumor progression [10, 11]. During EMT, epithelial cell lose its E-cadherin expression, which contributes to intercellular adhesion and enables cells with migratory and invasive properties. Increase expression of N-cadherin, Vimentin and Fibronectin can be markers for metastasis and poor prognosis [12–14]. EMT has been demonstrated to be a good target for various therapeutic interventions [15, 16].

In this study, we will compare expression of AFAP1-AS1 in CRC tissues and paired adjacent non-cancerous tissues for purpose of identification of abnormal expression of AFAP1-AS1 in relation to tumorigenesis of CRC. The potential mechanism by which AFAP1-AS1 regulates tumor progress was determined in basis of in vitro CRC cells culture.

## Methods

### Clinical tissues

Total 15 patients that have never received therapy before surgery were recruited from Affiliates Hospital of Beihua University in present study. Tissues were collected after acquirement of informed consent from patients. CRC tissues and paired adjacent normal tissues were collected and rapid frozen in liquid nitrogen for subsequent real time PCR analysis. All protocols have been approved by Ethics Committee of Affiliates Hospital of Beihua University.

### Colorectal carcinoma cell lines

Colorectal cancer cell lines HCT116 and SW480 were obtained from the ATCC and cultured in RPMI 1640 (Hyclone) with supplement of 10 % FBS (Gibco-BRL; Invitrogen) at a humidity of 5 % CO_2_ at 37 °C.

### Quantitative real time PCR

Total RNA was extracted from colorectal tissues of CRC patients or CRC cells with Trizol Reagent (Qiagen, Hilden). Complementary DNA (cDNA) was synthesized with equal 1 μg of total RNA by using superscript III reverse transcriptase (Invitrogen Life Technologies, Carlsbad, CA). One microliter of the cDNA was amplified by real time PCR for AFAP1-AS1 using Bio-Rad iCycler iQ Real-Time PCR Detection System (Bio-Rad Laboratories, Inc., Hercules, CA) with SYBR Green qPCR Super Mix (Invitrogen, Carlsbad, CA). Relative expression was calculated with the comparative Ct method and normalized against the Ct of β-Actin. The primers for qRT-PCR are as follows:AFAP1-AS1, Forward 5′-TCGCTCAATGGAGTGACGGCA-3′; Reverse 5′-CGGCTGAGACCGCTGAGAACTT-3′; AFAP1, Forward 5′-AGAGTGTCCTCCTCCACCAA-3′ Reverse 5′-CTTGGCCTCTGATTTGGAAC-3′ GAPDH, Forward 5′- CACCCACTCCTCCACCTTTG-3′; Reverse 5′-CCACCACCCTGTTGCTGTAG-3′.

### Si-AFAP1-AS1 transfection

To knockdown AFAP1-AS1, the siRNA oligonucleotides targeting AFAP1-AS1 and control (as mock indicated in manuscript) transfected into SW480 cells using Lipofectamine RNAiMAX (Invitrogen, CA) according to the manufacturer’s protocols. The sequences of the AFAP1-AS1 targeting siRNAs were 5′-GGGCTTCAATTTACAAGCATT-3′ (Si-RNA1) and 5′-CCTATCTGGTCAACACGTATT-3′ (Si-RNA 2).

### MTT assay

CRC cell proliferation was evaluated by MTT ([3-(4,5-dimethylthiazol-2-yl)-2,5 -diphenyltetrazolium bromide) assay. In brief, 1 × 10^4^ cell/well cells were seeded in triplicate in 48-well plates and cultured in normal condition for 24 h under. Cells were transfected with si-AFAP1-AS1 for 24 h for subsequent MTT assay with MTT cell proliferation assay kit (Abnova, Walnut, CA), according to manufacturer’s protocols. Absorbance of each well was measured at 570 nm with a spectrophotometer (ELISA reader, Dynatech Laboratories, Chantilly, VA).

### Colony formation

Cell in logarithmic phase was digested with 0.25 % trypsin and suspended in RPMI 1640 medium. A total of 400 cells were uniformly inoculated into 10 ml petri dish and cultured in normal condition for 2 weeks till visible colon. Colons were then fixed with 4 % paraformaldehyde for 15 min for subsequent staining with GIMSA. Colony number was calculated under optical microscope.

### Wound scratch assay

A total of 5 × 10^5^ cells were seeded in six-well culture plates and grown to 85–90 % confluence. Scratch was then vertically created by a p200 pipet tip in the cell monolayer. After a period of 24 h incubation in normal condition, images for cell growth around wounding were captured. Number of cell number around wounding calculated and was normalized to Mock.

### Transwell invasion assay

SW480 cell invasion capacity was assessed using Transwell Chamber Cell Culture (10-μm pore membrane, BD Biosciences). A total of 1 × 10^5^ cells in 100 μl of serum-free medium were added to the top chamber of 24-well plates that has been pre-added with Matrigel. The bottom well contained growth medium with 20 % FBS. Transwell chambers were placed at 37 °C for 36 h. Cells in chamber were fixed with methanol for 30 min and then staining with Giemsa for 15–30 min. Invaded cells were finally observed under a microscope and the number was counted with randomly nine field for each experiment.

### Western blot

Protein expression of N-cadherin, vimentin, Fibronectin E-cadherin and MMP-9 was performed with immunoblot analysis in cell lysates. After total protein quantification by BCA kit, an equal quantity 30–40 μg protein of all samples was separated by 10 % sodium dodecyl sulfate polyacrylamide gel electrophoresis (SDS–PAGE), and then electrophoretic transferred onto PVDF membrane. Membranes were experienced blockage with 5 % skim milk in TBS buffer, incubation with primary antibodies for target gene, and subsequent incubation with horseradish peroxidase-conjugated secondary antibody. Between each step, membranes was washed with TBS suppled with 20 % Tween20. In the present experiment, anti-E-cadherin antibody, anti-N-cadherin antibody, anti-Vimentin antibody, anti-Fibronectin antibody and anti-MMP9 antibody were purchased from Cell Signaling Technology (Danvers, MA, USA); anti-GAPDH antibody and anti-AFAP1 antibody were purchased from Proteintech (Wuhan, China). The signal was finally visualized using an ECL detection reagent on Bio-Rad ChemiDoc MP. Visualization of GAPDH expression was used as a loading control.

### Xenograft

Male C57BL/6 nude mice with 7-week-age were obtained from SLAC Laboratory Animals Co Ltd (Shanghai, China). Animals protocols have been approved by animal Ethics Committee of Affiliates Hospital of Beihua University and performed according to the institutional guidelines.

Subcutaneous tumor transplantation was performed by 10^6^ of SW480 cells injection in the right flank of mice. Tumor was removed after 4 weeks transplantation and tumor weight was measured.

Hepatic metastasis of colonic carcinoma was performed by 10^6^ of SW480 cells injection into spleen of nude mice. Mice was euthanatized after 4 weeks injection. Hepatic metastasis nodules were then counted.

### Statistical analysis

Statistical analysis was performed using SPSS software (version 16.0; Chicago, IL). Student’s t tests were used to evaluate significant differences between any two groups of data, or one-way analysis of variance with the Bonferroni post-test was performed for three or more comparisons. All data are represented as mean ± standard deviation. A p values <0.05 was considered significant.

## Results

### Abnormal expression of AFAP1-AS1 in colorectal carcinoma

**Fig. 1:**
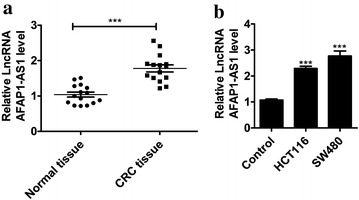
Relative expression of AFAP1-AS1 in **a** colorectal carcinoma (CRC) tissues (n = 15) and **b** colorectal carcinoma HCT116 and SW480 cell lines. ^***^P < 0.001 compared with normal tissues (paired para-carcinoma tissue) or normal colorectal cells

To determine the correlation between abnormal expression of AFAP1-AS1 and tumorigenesis of colorectal carcinoma, we examined relative expression of AFAP1-AS1 in 15 clinical CRC tissues with paired para-carcinoma tissue as control. As shown in Fig. 1a, AFAP1-AS1 expressed significantly higher in CRC tissues than normal tissues. Moreover, relative expression of AFAP1-AS1 was also elevated in CRC cells lines of HCT116 and SW480 (Fig. 1b).

### Proliferation of colorectal carcinoma in AFAP1-AS1 knock-down CRC cells

**Fig. 2:**
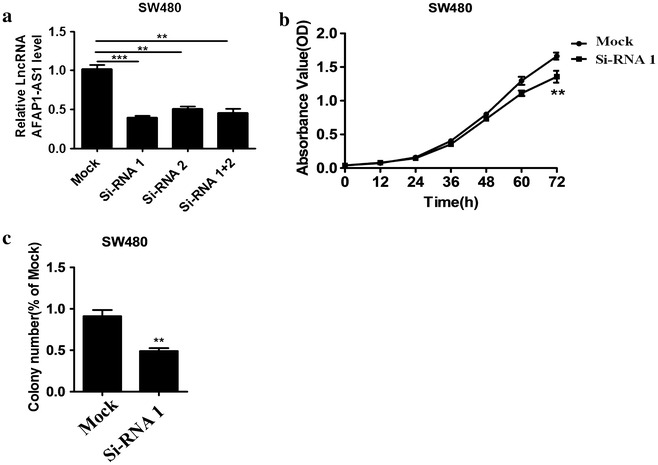
Effect of AFAP1-AS1 knock-down on proliferation of colorectal carcinoma. **a** Si-AFAP1-AS1 including si-RNA1 and si-RNA2 transfection contributes downregulation of AFAP1-AS1 in comparison to mock (as control). **b** Cell proliferation by MTT assay and **c** colony formation were determined in si-RNA1-transfected SW480 cells. ^**^P < 0.01, ^***^P < 0.001 compared with mock

To explore the role of AFAP1-AS1 on CRC cell proliferation, we examined cell proliferation in si-RNA1 or siRNA-2 transfected SW480. Si-AFAP1-AS1 transfection contributed to 5.9-fold decline of relative expression of AFAP1-AS1 in comparison to mock (Fig. 2a). This AFAP1-AS1 knock-down timely suppressed cell proliferation indicated by reduced OD values in si-RNA1-treated cells by MTT assay (Fig. 2b). In addition, AFAP1-AS1 knock-down suppressed colony formation that substantially reduced colony number (Fig. 2c).

### Metastasis of colorectal carcinoma in AFAP1-AS1 knock-down CRC cells

**Fig. 3:**
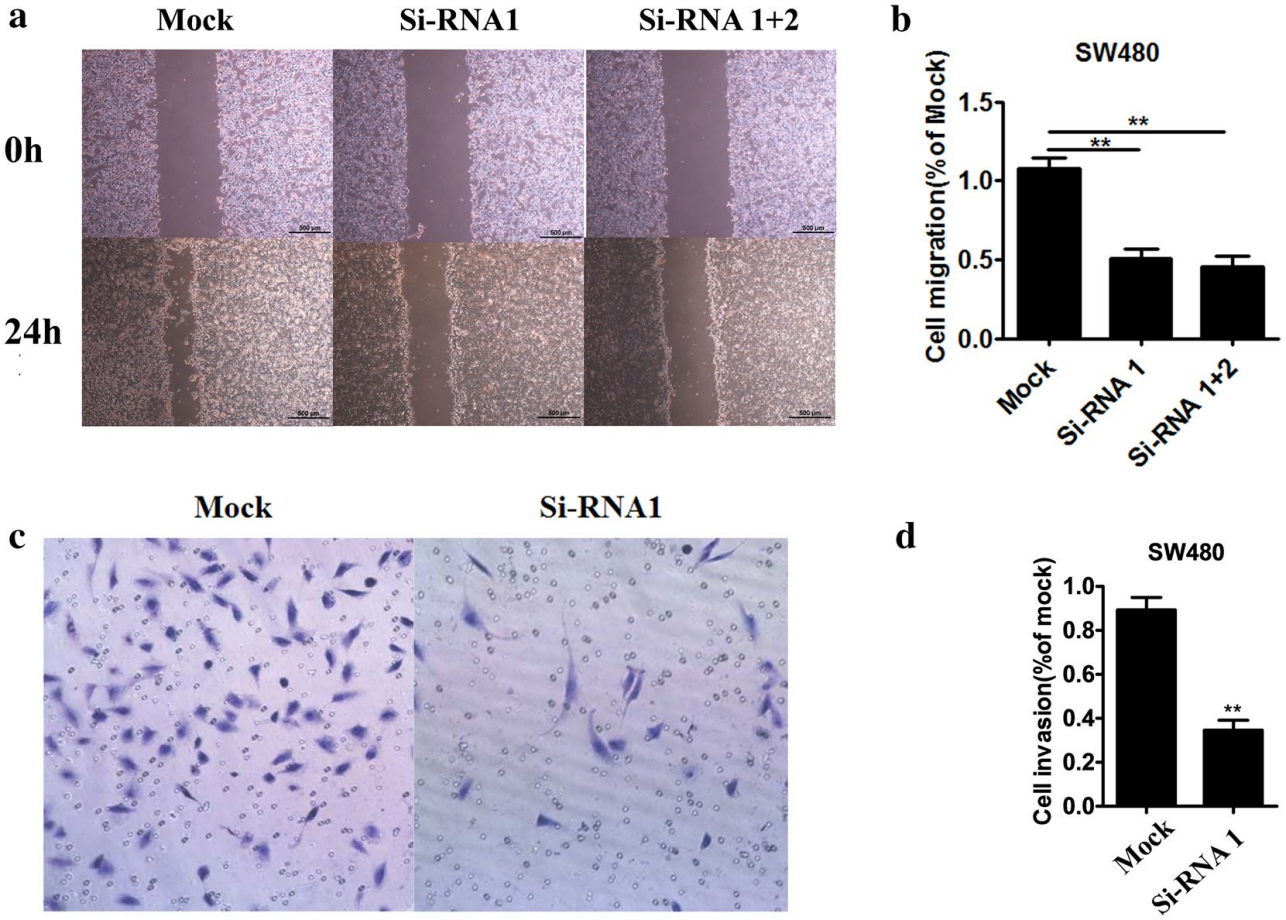
Effect of AFAP1-AS1 knock-down on metastasis of colorectal carcinoma. **a** Image presents cell wound scratch assay. **b** Relative migrated cell was normalized to mock treated cells. **c** Image presents cell invasion. **d** Relative invasive cell was normalized to mock treated cells. ^**^P < 0.01 compared with mock

We next examined CRC cell migration and cell invasion in si-AFAP1-AS1 transfected SW480. Wound scratch assay was performed to gauge the effect of AFAP1-AS1 knock-down on cell motility in Fig. 3a which showed wound recovery was significantly delayed in AFAP1-AS1-specific siRNA-treated SW480 cells. The migrated cell was calculated and normalized to mock (Fig. 3b). Cell metastasis was assessed using invasion assays as shown in Fig. 3c in which cell migration was suppressed in AFAP1-AS1 knock-down SW480 cells. Cell migration was reduced by 51 % (Fig. 3d).

### Inhibition of AFAP1-AS1 in change of EMT associated gene expression

**Fig. 4:**
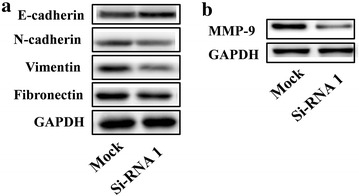
AFAP1-AS1 knockdown promotes EMT. Western blot analysis determined expression **a** E-cadherin, N-cadherin, vimentin, Fibronectin and **b** MMP-9 in si-RNA1-transfected SW480 cells

Epithelial to mesenchymal transition (EMT) is a tumor promotion process that mediates tumor invasion and metastasis. We performed western blot to examine change of EMT associated gene expression in AFAP1-AS1-specific siRNA-treated SW480 cells in Fig. 4a which showed elevated E-cadherin and reduced N-cadherin, Vimentin and Fibronectin by si-AFAP1-AS1. In addition, expression of matrix metalloproteinase (MMP) was inhibited (Fig. 4b).

### Xenograft growth of AFAP1-AS1 knock-down SW480 cell

**Fig. 5:**
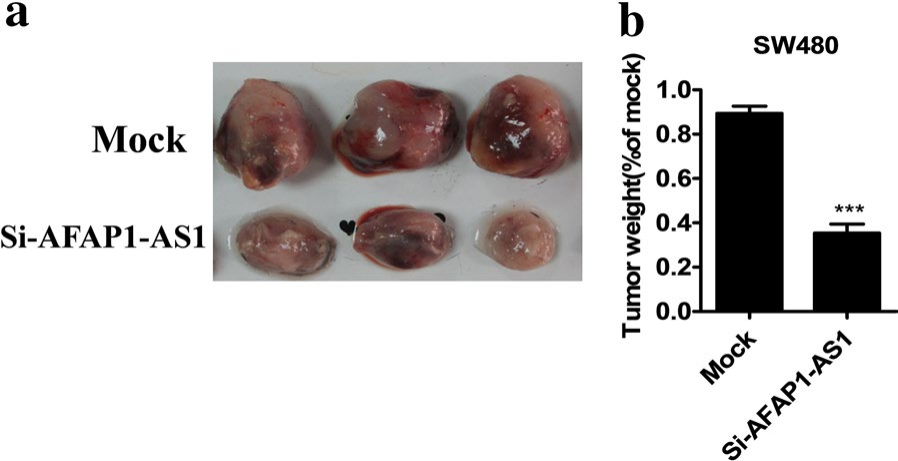
Effect of AFAP1-AS1 knock-down on xenograft growth. SW480 that has been transfected with si-AFAP1-AS1 or mock was subcutaneously injected nude mice. **a** Images presents tumor after 4 weeks transplantation. **b** Tumor weight was normalized to mock. ^***^P < 0.001 compared with mock

To investigate the function involvement of AFAP1-AS1 in growth of CRC cell xenograft, we subcutaneously transplanted the AFAP1-AS1-specific siRNA-treated SW480 cells to nude mice for purpose of examination of tumor growth. Morphologic observation in Fig. 5a showed smaller xenograft of AFAP1-AS1 knock-down SW480 cell than mock xenograft. By measurement of tumor weight, we observed that AFAP1-AS1 knock-down effectively reduced 5.2-fold of tumor weight (Fig. 5b).

### Hepatic metastasis of colonic carcinoma in AFAP1-AS1 knock-down SW480 cell

**Fig. 6:**
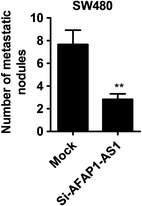
Effect of AFAP1-AS1 knock-down on hepatic metastasis of colonic carcinoma. SW480 that has been transfected with si-AFAP1-AS1 or mock was injected into spleen of nude mice. Number of metastasis nodules was counted after 4 weeks injection. ^**^P < 0.01 compared with mock

To investigate the function involvement of AFAP1-AS1 in hepatic metastasis of colonic carcinoma, we injected the AFAP1-AS1-specific siRNA-treated SW480 cells to spleen of nude mice for purpose of examination of number of metastasis nodules. Counting metastasis nodules was showed in Fig. 6a, in which AFAP1-AS1 knock-down caused decreased number of metastasis nodules compared with mock.

### Lnc-AFAP1-AS1 negatively regulates AFAP1 expression

**Fig. 7:**
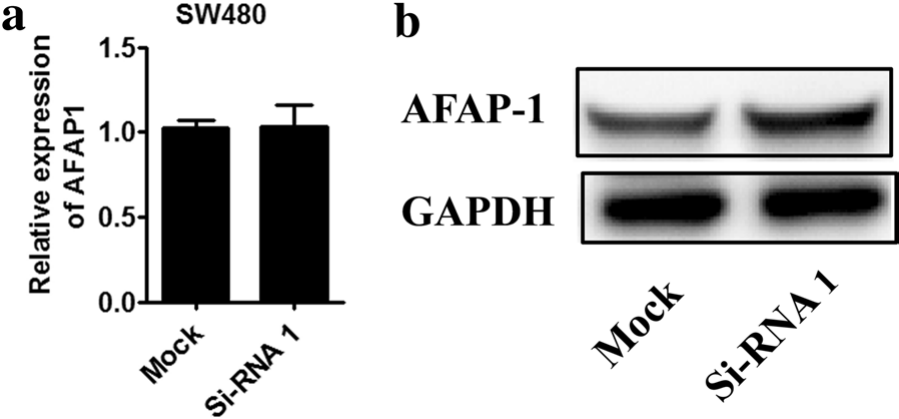
AFAP1-AS1 knock-down affects AFAP1 expression. **a** Relative expression of AFAP1 mRNA and **b** western blotting of AFAP1 protein after si-RNA1 transfection were determined in si-RNA1

We next detected the effect of lncRNA-AFAP1-AS1 on mRNA and protein level of AFAP1. As in Fig. 7, knockdown Lnc-AFAP1-AS1 could promote the protein level of AFAP1 while having no effect on the mRNA level of AFAP1.

## Discussion

We have set up a protocol to identify functional involvement of lncRNA-AFAP1-AS1 in CRC tumorigenesis. Our data suggested that tissues and CRC cells expressed high level of AFAP1-AS1 and this upregulation correlated with CRC cell proliferation and metastasis. Previous studies have been suggested that AFAP1-AS1 serves as a promotor for other cancers. This present study was the first to define AFAP1-AS1 as a tumor promotor in CRC.

AFAP1-AS1 is recognized in recent years and was originally found in esophageal adenocarcinoma [8]. Since then researchers further explored its anti-cancer roles in other cancer, such as pancreatic ductal adenocarcinoma, nasopharyngeal carcinoma and hepatocellular carcinoma etc. [7, 17, 18], in which upregulation of AFAP1-AS1 was associated with cancer poor prognosis and oncogenesis. In keeping with these previous studies, our present study showed that AFAP1-AS1 had the higher fold change in CRC tissues compared with paired adjacent normal tissues. We thus assessed AFAP1-AS1 function for CRC progression in CRC SW480 cells transfected with AFAP1-AS1-specific siRNA. Similar to expression in CRC tissues, AFAP1-AS1 was also enhanced in CRC HCT116 cells and SW480 cells. By AFAP1-AS1-specific siRNA (si-AFAP1-AS1) transfection, AFAP1-AS1 expression can reduce 56 % of mock treated SW480, and this AFAP-AS1 silencing resulted in suppression of CRC cell proliferation, indicating by MTT assay and colony formation assay. Anti-proliferation, a process that constituted basis of tumor growth by AFAP1-AS1 has been proved in previous study [19]. This data suggested anti-tumor formation action of AFAP1-AS1 silencing.

Our in vitro experiments also demonstrated that AFAP1-AS1 knockdown resulted in suppression of migration and invasion ability of CRC SW480 cells. Tumor metastasis is a multi-step process by which tumor cells disseminate form their primary site and form secondary tumor at a distant site. EMT is such a pivotal and intricate mediator in this process. The initially step of EMT process is E-cadherin suppression, thereby reduce cell adhesion. Our data showed that AFAP1-AS1 knockdown caused enhancement of E-cadherin expression, providing an evidence of anti-metastasis action of AFAP1-AS1. Other migration related proteins that control the balance between cell–cell and cell-extracellular matrix adhesion, such as N-cadherin, Vimentin and Fibronectin [20], were also been positively regulated by AFAP1-AS1 silencing. Metalloproteinase-9 (MMP-9) belongs to a family of proteolytic enzymes that catalyze extracellular matrix (ECM) degradation and has a critical role in several stages of tumor progression [21]. Tumor metastasis commonly accompanied by increasing expression of MMP-9. We therefore examined the expression of MMP-9 in AFAP1-AS1 knock-down SW480 cells and observed downregulation of MMP-9. These data further confirmed the pro-metastasis role of AFAP1-AS1.

The anti-tumor action of AFAP1-AS1 silencing was confirmed by in vivo mice-bearing tumor experiments. AFAP1-AS1-specific siRNA-treated SW480 cells were transplanted in the right flank or spleen of mice to evaluate tumor growth and hepatic metastasis. Tumor formation ability of AFAP1-AS1 silenced SW480 cells was significantly suppressed. Also hepatic metastasis ability of colonic carcinoma was substantially attenuated. In addition, we found that AFAP1-AS1 silencing could promote the expression level of actin filament-associated protein 1 (AFAP1) protein, a binding partner for oncogenic Src [22], while having no effect on the level of AFAP1 mRNA. Our results are consistent with the previous study by Hao Bo et al. [17]. In this study, they found that AFAP1-AS1 knockdown could induce the loss of stress filament integrity and affect the expression of Rho/Rac GTPase family members and actin cytokeratin signaling pathway proteins in nasopharyngeal cancer cells, so we speculate that AFAP1-AS1 knockdown could also affect actin cytoskeleton via small Rho GTPase in colorectal cancer cell, we will confirm it in the further study.

In conclusion, our data suggested that lncRNA-AFAP1-AS1 was the tumor promotor of CRC and was associated with CRC cell proliferation and migration thereby contributes to CRC tumor growth and metastasis. The pro-cancer action of AFAP1-AS1 might be mediated by EMT process.

## References

[1] Carroll MR, Seaman HE, Halloran SP (2014). Tests and investigations for colorectal cancer screening. Clin Biochem.

[2] Sag AA, Selcukbiricik F, Mandel NM (2016). Evidence-based medical oncology and interventional radiology paradigms for liver-dominant colorectal cancer metastases. World J Gastroenterol.

[3] Wu R, Su Y, Wu H, Dai Y, Zhao M, Lu Q (2016). Characters, functions and clinical perspectives of long non-coding RNAs. Mol Genet Genomics..

[4] Han D, Wang M, Ma N, Xu Y, Jiang Y, Gao X (2015). Long noncoding RNAs: novel players in colorectal cancer. Cancer Lett.

[5] Ma C, Shi X, Zhu Q, Li Q, Liu Y, Yao Y, Song Y (2016). The growth arrest-specific transcript 5 (GAS5): a pivotal tumor suppressor long noncoding RNA in human cancers. Tumour Biol.

[6] Ye LC, Ren L, Qiu JJ, Zhu DX, Chen T, Chang WJ, Lv SX, Xu J (2015). Aberrant expression of long noncoding RNAs in colorectal cancer with liver metastasis. Tumour Biol.

[7] Ye Y, Chen J, Zhou Y, Fu Z, Zhou Q, Wang Y, Gao W, Zheng S, Zhao X, Chen T (2015). High expression of AFAP1-AS1 is associated with poor survival and short-term recurrence in pancreatic ductal adenocarcinoma. J Trans Med.

[8] Wu W, Bhagat TD, Yang X, Song JH, Cheng Y, Agarwal R, Abraham JM, Ibrahim S, Bartenstein M, Hussain Z (2013). Hypomethylation of noncoding DNA regions and overexpression of the long noncoding RNA, AFAP1-AS1, Barrett’s esophagus and esophageal adenocarcinoma. Gastroenterology.

[9] Zeng Z, Bo H, Gong Z, Lian Y, Li X, Li X, Zhang W, Deng H, Zhou M, Peng S (2016). AFAP1-AS1, a long noncoding RNA upregulated in lung cancer and promotes invasion and metastasis. Tumour Biol.

[10] Costabile V, Duraturo F, Delrio P, Rega D, Pace U, Liccardo R, Rossi GB, Genesio R, Nitsch L, Izzo P (2015). Lithium chloride induces mesenchymaltoepithelial reverting transition in primary colon cancer cell cultures. Int J Oncol.

[11] Li Y, Dong M, Kong F, Zhou J (2015). Octamer transcription factor 1 mediates epithelial-mesenchymal transition in colorectal cancer. Tumour Biol.

[12] Ye Z, Zhou M, Tian B, Wu B, Li J (2015). Expression of lncRNA-CCAT1, E-cadherin and N-cadherin in colorectal cancer and its clinical significance. Int J Clin Exp Med.

[13] Wang H, Wang HS, Zhou BH, Li CL, Zhang F, Wang XF, Zhang G, Bu XZ, Cai SH, Du J (2013). Epithelial-mesenchymal transition (EMT) induced by TNF-alpha requires AKT/GSK-3beta-mediated stabilization of snail in colorectal cancer. PLoS One.

[14] Xiao S, Liu L, Lu X, Long J, Zhou X, Fang M (2015). The prognostic significance of bromodomain PHD-finger transcription factor in colorectal carcinoma and association with vimentin and E-cadherin. J Cancer Res Clin Oncol.

[15] Chen ZJ, Wei W, Jiang GM, Liu H, Wei WD, Yang X, Wu YM, Liu H, Wong CK, Du J (2016). Activation of GPER suppresses epithelial mesenchymal transition of triple negative breast cancer cells via NF-kappaB signals. Mol Oncol..

[16] Li CW, Xia W, Lim SO, Hsu JL, Huo L, Wu Y, Li LY, Lai CC, Chang SS, Hsu YH (2016). AKT1 inhibits epithelial-to-mesenchymal transition in breast cancer through phosphorylation-dependent Twist1 degradation. Cancer Res.

[17] Bo H, Gong Z, Zhang W, Li X, Zeng Y, Liao Q, Chen P, Shi L, Lian Y, Jing Y (2015). Upregulated long non-coding RNA AFAP1-AS1 expression is associated with progression and poor prognosis of nasopharyngeal carcinoma. Oncotarget.

[18] Zhang JY, Weng MZ, Song FB, Xu YG, Liu Q, Wu JY, Qin J, Jin T, Xu JM (2016). Long noncoding RNA AFAP1-AS1 indicates a poor prognosis of hepatocellular carcinoma and promotes cell proliferation and invasion via upregulation of the RhoA/Rac2 signaling. Int J Oncol.

[19] Lu X, Zhou C, Li R, Liang Z, Zhai W, Zhao L, Zhang S. Critical role for the long non-coding RNA AFAP1-AS1 in the proliferation and metastasis of hepatocellular carcinoma. Tumour Biol. 2016. 10.1007/s13277-016-4858-8 PMC4990603 26803513

[20] Broders-Bondon F, Paul-Gilloteaux P, Gazquez E, Heysch J, Piel M, Mayor R, Lambris JD, Dufour S (2016). Control of the collective migration of enteric neural crest cells by the Complement anaphylatoxin C3a and N-cadherin. Dev Biol..

[21] Wu X, Liu BJ, Ji S, Wu JF, Xu CQ, Du YJ, You XF, Li B, Le JJ, Xu HL (2015). Social defeat stress promotes tumor growth and angiogenesis by upregulating vascular endothelial growth factor/extracellular signal-regulated kinase/matrix metalloproteinase signaling in a mouse model of lung carcinoma. Mol Med Rep.

[22] Cho Y, Silverstein R, Geisinger MT, Martinkovich S, Corkill H, Cunnick JM, Planey SL, Arnott JA (2015). AFAP1 is a novel downstream mediator of TGF-beta1 for CCN2 induction in osteoblasts. PLoS One.

